# Microhabitat Conditions in Wyoming’s Sage-Grouse Core Areas: Effects on Nest Site Selection and Success

**DOI:** 10.1371/journal.pone.0150798

**Published:** 2016-03-22

**Authors:** Jonathan B. Dinkins, Kurt T. Smith, Jeffrey L. Beck, Christopher P. Kirol, Aaron C. Pratt, Michael R. Conover

**Affiliations:** 1 Department of Ecosystem Science and Management, University of Wyoming, Laramie, Wyoming, United States of America; 2 Department of Wildland Resources, Utah State University, Logan, Utah, United States of America; University of Regina, CANADA

## Abstract

The purpose of our study was to identify microhabitat characteristics of greater sage-grouse (*Centrocercus urophasianus*) nest site selection and survival to determine the quality of sage-grouse habitat in 5 regions of central and southwest Wyoming associated with Wyoming’s Core Area Policy. Wyoming’s Core Area Policy was enacted in 2008 to reduce human disturbance near the greatest densities of sage-grouse. Our analyses aimed to assess sage-grouse nest selection and success at multiple micro-spatial scales. We obtained microhabitat data from 928 sage-grouse nest locations and 819 random microhabitat locations from 2008–2014. Nest success was estimated from 924 nests with survival data. Sage-grouse selected nests with greater sagebrush cover and height, visual obstruction, and number of small gaps between shrubs (gap size ≥0.5 m and <1.0 m), while selecting for less bare ground and rock. With the exception of more small gaps between shrubs, we did not find any differences in availability of these microhabitat characteristics between locations within and outside of Core Areas. In addition, we found little supporting evidence that sage-grouse were selecting different nest sites in Core Areas relative to areas outside of Core. The Kaplan-Meier nest success estimate for a 27-day incubation period was 42.0% (95% CI: 38.4–45.9%). Risk of nest failure was negatively associated with greater rock and more medium-sized gaps between shrubs (gap size ≥2.0 m and <3.0 m). Within our study areas, Wyoming’s Core Areas did not have differing microhabitat quality compared to outside of Core Areas. The close proximity of our locations within and outside of Core Areas likely explained our lack of finding differences in microhabitat quality among locations within these landscapes. However, the Core Area Policy is most likely to conserve high quality habitat at larger spatial scales, which over decades may have cascading effects on microhabitat quality available between areas within and outside of Core Areas.

## Introduction

Quantity and quality of breeding habitat have been suggested as the most important factors dictating the productivity of greater sage-grouse (*Centrocercus urophasianus*; hereafter, sage-grouse) populations [1,2,3]. Studies have reported that sage-grouse select nest sites based on a preference for different microhabitat characteristics, such as sagebrush density [4,5], sagebrush cover [6,7], shrub height [8], grass height [7,8,9], and grass cover [7,10]. These studies all indicated that sage-grouse choose nest locations in habitats with greater concealment cover. However, there are differences in the quality of local microhabitat available as nesting habitat for female sage-grouse across Wyoming.

While sage-grouse select for microhabitat characteristics that provide greater concealment cover from predators and protection from weather, selection of habitat is limited to the range of microhabitat conditions that are available. Microhabitat characteristics around a nest, such as sagebrush cover and grass height, can facilitate or impede a predator from depredating a nest [11,12], and this varies among different types of nest predators [11,13]. Sage-grouse consistently select for greater sagebrush cover [7,9,14,15,16]; however, the connection between sagebrush cover and nest success has been more variable with many studies failing to find a relationship between nest success and sagebrush cover with some exceptions [8,17,18]. The current knowledge of sage-grouse nesting ecology indicates that sagebrush (or shrub) cover is important, but the effect of sagebrush cover on nest success among local areas is variable. Holloran et al. [9] suggested that within patch scales, sagebrush metrics remain relatively constant throughout and among breeding seasons, whereas, grass cover and height were more variable and dependent on weather conditions. A study in southeast Montana and northeast Wyoming found that grass height variability influenced by spring precipitation was highly predictive of nest success in sage-grouse [19]. Habitat quality is highly variable and depends on multiple environmental and anthropogenic factors. Variability in the condition of available microhabitat throughout sage-grouse range may significantly influence specific life-history stages and associated vital rates (e.g., nest success). Therefore, studies across broader ranges and more diverse microhabitat have the greatest potential to identify microhabitat variables that influence nest selection and success of sage-grouse at regional scales.

As a result of the 2008 Wyoming Governor’s Sage-grouse Executive Order, the State of Wyoming implemented the Core Area Policy to conserve sage-grouse populations in Wyoming [20]. This policy focuses on minimizing impacts to the highest quality sage-grouse habitats [21,22]. We have compiled an expansive dataset designed to evaluate sage-grouse nest site selection and nest success, where data were collected from multiple regions throughout Wyoming starting in the initial year of the Core Area Policy. The purpose of our study was to use microhabitat data collected over a broad range of sagebrush habitats in Wyoming to 1) identify microhabitat characteristics that influence sage-grouse selection of nest sites, 2) compare available microhabitat within and outside of Core Areas, 3) compare microhabitat used by sage-grouse for nesting between areas protected and not protected under the Core Area Policy, and 4) evaluate the influence of microhabitat on nest success and compare nest success within and outside of Core Areas. Our analysis contains an evaluation of several Core and non-Core Areas and distinct sage-grouse populations within Wyoming.

## Study Areas

Wyoming big sagebrush (*Artemisia tridentata wyomingensis*) at lower elevations and mountain big sagebrush (*A*. *t*. *vaseyana*) at higher elevations dominated our study areas [23]. Black sagebrush (*A*. *nova*) and/or low sagebrush (*A*. *arbuscula*) were found on exposed ridges. Other shrub species common to various study areas included alderleaf mountain mahogany (*Cercocarpus montanus*), antelope bitterbrush (*Purshia tridentata*), chokecherry (*Prunus virginiana*.), common snowberry (*Symphoricarpos albus*), greasewood (*Sarcobatus vermiculatus*), and rabbitbrush (*Chrysothamnus* and *Ericameria* spp.). Isolated stands of juniper (*Juniperus* spp.) and quaking aspen (*Populus tremuloides*) were found at higher elevations. Data within our study areas were collected 2008–2014, but active data collection differed for some of the study areas (Table 1). Annual precipitation within study areas ranged from approximately 22.3 cm to 49.3 cm (mean = 32.5 cm [2.7 SE]). All study areas had some anthropogenic features. In the less disturbed study areas this mainly consisted of unimproved 4-wheel drive roads. Oil and gas extraction activities occurred in each of the study areas (range 0.004–0.236 wells per km^2^) and consisted of conventional natural gas, coalbed methane natural gas, and/or conventional oil with an average of 0.18 per km^2^ outside Core Areas and 0.01per km^2^ within Core Areas. Livestock grazing by domestic sheep and cattle was a primary land use in all of the study areas.

**Table 1:** Summary of nest and random location sample sizes used for occurrence, availability (random-random), and nest success analyses, in central and southwestern Wyoming, USA, 2008–2014.

Sage-grouse study area	Years	Nests	Random
Atlantic Rim	2008–2011	123	122
Bighorn Basin	2011–2014	291	290
Jeffrey City	2011–2014	270	166
Southwest Wyoming	2008–2011	193	190
Stewart Creek	2008–2011	51	51
Total of all studies	2008–2014	928	819

### Atlantic Rim

**Fig 1:**
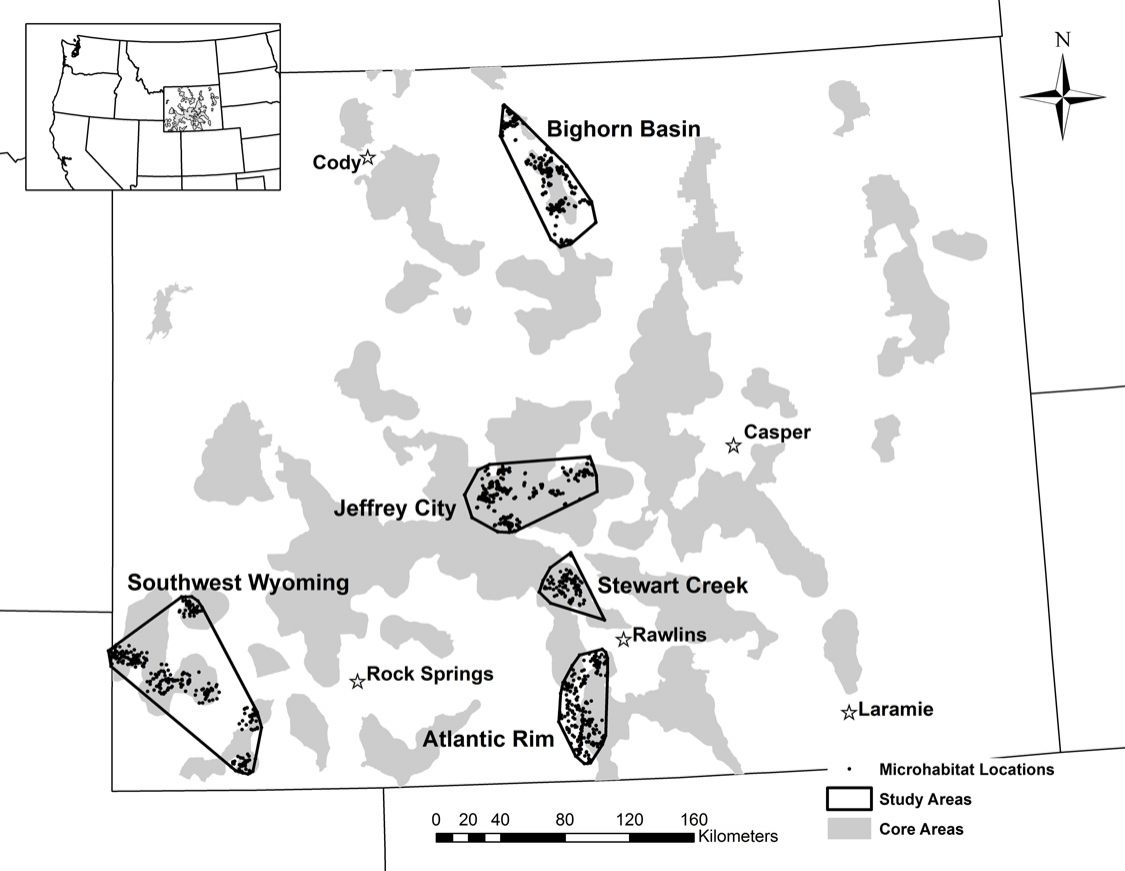
Map of study areas and microhabitat sampling points across five regions in central and southwestern Wyoming, USA, 2008–2014. Boundaries for each study area were demarcated by a 100% minimum convex polygon encompassing 928 sage-grouse nests and 819 random locations. However, the southwest Wyoming minimum convex polygon was an aggregation of 8 separate 8-km areas around sage-grouse leks. Locations of 31 sage-grouse Core Areas are also illustrated in gray.

The Atlantic Rim study area was located in southern Carbon County, Wyoming, extending approximately 77 km north and south between Rawlins and Baggs (Fig 1). This study area was located within and adjacent to the South Rawlins and Greater South Pass Core Areas. The area included approximately 64% federal, 5% state, and 31% private lands. The Atlantic Rim is within the semi-desert grass-shrub zone and is characterized by sagebrush steppe with low average annual precipitation. Elevation within the study area ranged from 1,982 to 2,529 m. Major land uses included oil and gas extraction.

### Bighorn Basin

The Bighorn Basin study area was located in Big Horn and Washakie counties, Wyoming (Fig 1). This study area was located within and adjacent to the Hyattville, Shell, and Washakie Core Areas. The area extends northeast of Greybull, Wyoming and south to near Ten Sleep, Wyoming in the eastern Bighorn Basin on the west slope of the Bighorn Mountains. The area included approximately 74% federal, 7% state, and 19% private lands. Elevation within the study area ranged from 1,300 to 2,850 m. Major land uses in this area included bentonite mining.

### Jeffrey City

The Jeffrey City study area was in portions of Fremont and Natrona counties, Wyoming (Fig 1). This study area was located within and adjacent to the Greater South Pass Core Area. The area included approximately 81% Federal, 7% State, and 12% privately administered lands. Elevation ranged from 1,642 to 2,499 m. There is interest to resume uranium mining, which historically was once a major land use in the area.

### Southwest Wyoming

The Southwest Wyoming study area consisted of eight distinct study sites that were approximately 16 km in diameter centered on leks; this distance was based on results found by Holloran and Anderson [24] that indicated 93% of 415 nests were within 8.5 km from leks in central and southwestern Wyoming (Fig 1). The Southwestern Wyoming study area was located within and adjacent to the Fontenelle, Sage, and Uinta Core Areas. Five study sites were located in Lincoln, one in Sweetwater, and two in Uinta counties, Wyoming. Study sites were chosen to provide a representation of overall sage-grouse nesting habitat in southwestern Wyoming. Elevation ranged from 1,925 to 2,550 m. The area included approximately 65% Federal, 4% State, and 31% privately administered lands. Oil and gas development was present in 37.5% of the study area.

### Stewart Creek

The Stewart Creek study area was located in northern Carbon County, approximately 32.2 to 64.4 km north and west of Rawlins, Wyoming (Fig 1). This study area was located within and adjacent to the Greater South Pass Core Area. The area includes approximately 70% federal, 5% state, and 25% privately administered lands. The Stewart Creek area is within the semi-desert grass-shrub zone, characterized by sagebrush steppe with low annual precipitation. Elevation ranged from 1,982 to 2,529 m.

## Methods

### Capture and monitoring

We captured and radio-marked female sage-grouse from leks in spring by spot-light and hoop-net methods [25,26] from 2008–2014. We also used roosting locations of radio-marked adult females captured in spring to capture and radio-mark additional females in late summer each year. We aged females as juveniles or adults based on the shape and condition of the outermost wing primaries, the outline of the primary tail feathers, and coloration of undertail coverts [27,28]. We attached radio transmitters (22 g, Model A4060; Advanced Telemetry Systems Incorporated, Isanti, MN, USA) to females with a PVC-covered wire necklace. In the Bighorn Basin and Jeffrey City study areas, we attached GPS transmitters (22-g Solar Argos/GPS PTT-100, Microwave Telemetry, Columbia, MD, USA or Model 22 GPS PTT, North Star Science and Technology, King George, VA, USA) via rump mounts instead of necklace radio collars to a small portion of captured females. These GPS transmitters were programmed to acquire 4 locations per day via satellite (Argos, www.argos-system.org) from 15 March to 30 April, and 5 locations per day from 1 May to 24 August. We located VHF-collared females weekly beginning mid-to-late April each year with R-1000 hand-held receivers and 3-element Yagi antennas (Communication Specialists, Orange, CA, USA). All sage-grouse were captured, marked, processed, and monitored in adherence with approved protocols (Atlantic Rim and Stewart Creek studies [Wyoming Game and Fish Department {WGFD} Chapter 33 permits 572 and 699 and University of Wyoming Institutional Animal Care and Use Committee {UW IACUC} protocol 03032009]; Bighorn Basin study [WGFD Chapter 33–800 permit and UW IACUC protocols 03142011 and 20140228JB00065]; Jeffrey City study [WGFD Chapter 33–801 permit and UW IACUC protocols 03132011 and 20140128JB0059]; Southwest Wyoming study [WGFD Chapter 33 permit 657 and Utah State University IACUC protocol 1357]).

We located nests by circling a radio-marked (necklace collars) female until the surveyor visually located the female under a shrub or isolated the hen’s location. To minimize human-induced nest depredation or nest abandonment, we subsequently monitored nests of radio-marked females with triangulation from a distance of at least 30 m. For GPS-equipped females, we visually inspected potential nests after the female left a location of clustered GPS points. Nest success (i.e., nests with at least 1 hatched egg) was determined by examining egg shells after the female was no longer located at the nest site.

### Microhabitat sampling

We sampled microhabitat at nest and random locations for all study areas in all years with the exception that no random sampling was conducted in Jeffrey City during 2014. In the Atlantic Rim, Southwest, and Stewart Creek study areas, random locations for comparison to nest locations were established throughout the study area within 8 km of all leks where sage-grouse capture was conducted with ArcMap. We used Northwest GAP land-cover data [29] to constrain random locations to sagebrush habitats while excluding areas of inappropriate habitat such as exposed rock, open water, and conifer stands. In the Bighorn Basin and Jeffrey City study areas, we constrained random sampling at a random distance and direction 0.1–0.5 km from each paired nest location [30]. We chose a simulated nest site at random locations by selecting the closest shrub taller than or equal to 30 cm [9,31].

We used established protocols to measure the vegetation characteristics of microhabitat surrounding nests and random locations [5]. We began sampling nest microhabitat plots after the first successful hatch and attempted to sample nest and random locations concurrently within 2 weeks of known nest fate. We measured microhabitat characteristics along two perpendicular 10-m transect lines centered on each nest or random location extending in each cardinal direction (Table 2). We recorded shrub canopy cover with the line intercept method [32,33]. We measured height (cm) at each shrub that intercepted the line. Average canopy cover and height were quantified as total shrub and big sagebrush species. Sagebrush cover and height calculations excluded mat-forming sub-shrub species (e.g., prairie sagewort [*A*. *frigida*]) that could not be considered concealment cover for nests. Average sagebrush and shrub cover were calculated at three microhabitat spatial scales by averaging values from the two perpendicular lines from the nest or random location to 1, 2.5, or 5 m away from the nest or random location. We estimated visual obstruction to the nearest 5 cm by visually estimating the closest point to the ground visible on a modified Robel pole from 5 m away from nest and random locations and 1-m above the ground [34,35]. We estimated cover of annual grasses, perennial grasses, residual perennial grasses, food forb cover (for list, *see* [7]), non-food forb cover, gravel and rock, bare soil, biological soil crust, and litter by recording cover in 6 cover classes within 20 x 50 cm quadrats (0.1 m^2^; [36]). Quadrats were placed along each transect line at the center of each transect (nest bowl or center of random location) and centered at 1 and 3 m from the transect intersection in each cardinal direction for a total of *n* = 9 quadrats at each microhabitat plot. Cover classes were demarcated as 1 = 0.1–1%, 2 = 1.1–5%, 3 = 5.1–25%, 4 = 25.1–50%, 5 = 50.1–75%, and 6 = 75.1–100%. Average cover for quadrat variables were calculated at three microhabitat spatial scales by averaging values from the inner 5 quadrats (transect center and quadrats place 1 m from the transect center; 1 m spatial scale), the outermost 4 quadrats (values describing average values 2.5 to 3.5 m [width of the quadrat frame] away from the nest or random location), and all 9 quadrats (3.5 m spatial scale). We measured the height of perennial and residual perennial grasses (cm) as the tallest portion of the plant (droop height) within 1 m of each quadrat.

**Table 2:** Descriptions of microhabitat variables used to evaluate selection, availability (random-random), and nest success of sage-grouse. Data were collected at 928 sage-grouse nests and 819 random locations in in 5 study areas in Wyoming, USA, 2008–2014.

Variable Name	Description
**Categorical Variables**	
Nest Shrub Spp.	Shrub species at nest or center of random plots. Classified as sagebrush or non-sagebrush
**Shrub Characteristics**	
Shrub^1^^,^^2^	Mean total shrub cover (%)
Artr^1^^,^^2^	Mean big sagebrush cover (%)
Shrub_H	Mean shrub height (cm)
Artr_H	Mean big sagebrush height (cm)
Gap^3^	Count of spaces between shrubs
VO	Mean visual obstruction (horizontal; cm) 5 m from plot location
**Grass Height**	
PerGrass_H	Mean maximum perennial droop height (cm)
ResGrass_H	Mean maximum residual droop height (cm)
**Herbaceous Canopy Cover (%)**^4^	
AnGrass^4^^,^^5^	Mean annual grass cover
PerGrass^4^^,^^5^	Mean perennial grass cover
ResGrass^4^^,^^5^	Mean residual grass cover
FoodF^4^^,^^6^	Mean food forb cover
NFoodF^4^^,^^6^	Mean non-food forb cover
**Ground Cover (%)**^**4**^	
BGround^4^^,^^7^	Mean bare cover
Cactus^4^	Mean cactus cover
BioCrust^4^	Mean biological soil crust cover
Rock^4^^,^^7^	Mean gravel and rock cover
Litter^4^	Mean litter cover

^1^Cover assessed at 1, 2.5, and 5 m away from transect center

^2^Proportion of Artr to Shrub assessed at 1, 2.5, and 5 m away from transect center ^3^Gap spacing was categorized as ≥0.5 m and <1.0 m (Gap_0.5m_), ≥1 m and < 2.0 m (Gap_1m_), ≥2.0 m and <3.0 m (Gap_2m_), and ≥3.0 m and <4.0 m (Gap_3m_) counted along transect lines

^4^Cover assessed at 1.5 and 3.5 m away from transect center and between 2.5–3.5 m away from transect center

^5^Variables combined to assess total grass cover (Grass)

^6^Variables combined to assess total forb cover (Forb)

^7^Variables combined to assess total bare, gravel and rock ground cover (BareRock)

Descriptions of shrub characteristics (sagebrush and total shrub cover, sagebrush and total shrub height, gaps in shrub cover, and visual obstruction), grass height (perennial and residual grass height), herbaceous canopy cover (annual, perennial, residual grass, and forb cover), and ground cover (bare ground, biological soil crust, rock, and litter cover) variables calculated from transect lines, Robel poles, and quadrats are provided in Table 2. In addition to measured shrub characteristics, we calculated the proportion of sagebrush to total shrub cover (ARTRpShr) at all microhabitat spatial scales, and we generated number of shrub cover gaps in four categories. Shrub cover gaps were classified as Gap_0.5m_ (≥0.5 m and <1.0 m), Gap_1m_ (≥1 m and < 2.0 m), Gap_2m_ (≥2.0 m and <3.0 m), or Gap_3m_ (≥3.0 m and <4.0 m), then the number of each gap classification was summed along transect lines. The proportion of sagebrush to total shrub within plots was intended to assess differences in shrub composition relative to sagebrush as a measure of shrub diversity between sage-grouse nests and random locations, within and outside of Core Areas for random and nest locations, and nest success. We also recorded the dominant shrub species at the center of nest and random locations, which was used as a categorical variable classifying the shrub directly above a nest or center of a random location as sagebrush or non-sagebrush.

### Data analysis

For all analysis types, we used an information theoretic approach for modeling [37]. All statistical analyses were conducted in R [38]. We compared models with Akaike’s information criterion corrected for small sample sizes (AIC_*c*_) and Akaike weights *w*_*i*_; [39] with function ‘model.sel’ in package MuMIn version 1.13.4 in R. We screened each variable to identify potentially informative variables, which were defined as variables with regression coefficient values with 85% confidence intervals (CIs) that did not overlap zero [40]. For variable screening, variables with 85% CIs that overlapped zero were eliminated from all further AIC_*c*_ modeling. We based our inference on regression models within 4 AIC_*c*_ of the top selected model [39]. For variables measured at multiple microhabitat spatial scales, we compared single variable models and only used the microhabitat spatial scales that were not correlated or the variable with the lowest AIC_*c*_ in additive models. Our modeling approach allowed us to use variables with the most predictive potential to make inferences about selection and nest success [41] at different microhabitat scales. Model averaging of coefficients and standard errors was employed when there was model uncertainty, which was defined as multiple competing models within 4 ΔAIC_*c*_ of the top model. For all competitive final models, we report 95% CI for parameter estimates and odds ratios, because we considered predictor variables with 95% CI not overlapping zero to be precise and predictor variables that had 95% CI that overlapped zero to be marginal. Thus, we focused our interpretations on the precise predictor variables.

All models included year (2008–2014) and the 30-year normal of annual precipitation as random effects to account for seasonal weather and overall habitat differences among nest and/or random locations across years and study areas. The 30-year normal of annual precipitation consistently accounted for more variation among sampling locations than study area; thus, we used the 30-year normal of precipitation as a random factor rather than study area. The 30-year normal of annual precipitation was extracted at a 1-km spatial resolution from PRISM [42]. Differences in sampling design for random locations were also accounted for with these random effects. To prevent multicollinearity, we did not include any two co-varying variables (|*r* ≥0.65|) in any model as determined by a Pearson’s correlation matrix. When variables were correlated, we included the variable with the lowest AIC_*c*_ in that model.

#### Selection and availability models

We used binomial generalized mixed models (GLMMs) to evaluate 1) nest selection of sage-grouse, 2) availability of microhabitat within and outside of Core Areas, and 3) to compare nest selection by sage-grouse within and outside of Core Areas. For ease of interpretability, we referred to these analysis types as 1) nest-random, 2) random-random, and 3) nest-nest. The nest-random analysis employed a use-availability design to evaluate sage-grouse nest site selection with binary logistic regression [43], whereas, the random-random and nest-nest analyses were intended to classify differences in microhabitat between locations (random or nest) within and outside of Core Areas.

We fitted GLMMs with year and 30-year normal of precipitation as random effects with function ‘lmer’ in package lme4 version 1.1–7 in R. Random locations were coded as the reference category for our nest-random analysis, random locations outside of Core Areas were coded as the reference category for our random-random analysis, and nest locations outside of Core Areas were coded as the reference category for our nest-nest analysis. Microhabitat variables considered as predictors included shrub characteristics, grass height, herbaceous canopy cover, and ground cover variables (Table 2). We compared all possible combinations of informative variables as additive models for each analysis type with AIC_*c*_ and *w*_*i*_. The interpretations of change in odds ratios (selection probabilities) per unit change in variables were calculated as the median change in odds bound by the range of variable values for that variable with the other variables in the model held at their mean value. We report means and SE for nest-random microhabitat data in S1 Table.

#### Nest success models

Nest success was evaluated with a mixed effects version of the Cox proportional hazard (Cox PH) model using function ‘coxme’ in package coxme version 2.2–4 in R. We used Cox PH to identify relationships between microhabitat predictor variables and nest success. Analysis of nest success was based on time-to-event data, and Cox PH models are commonly used to analyze time-to-event survival data [44]. The risk of failure (hazard ratio) is a function of the non-parametric baseline hazard, and the parametric predictor variables affecting failure [45]. Thus, beta estimates were presented as the risk of a nest failing with positive beta values indicating a variable was positively related to a greater risk of nest failure. In Cox PH models, the baseline hazard is assumed to have a proportional hazard of failure over time (proportional hazard assumption) for all predictor variables [45]. Thus, we tested the proportional hazard assumption for each predictor variable with the function ‘cox.ph’ in the coxme package in R [46]. As in our GLMMs, we fit Cox PH models with year and 30-year normal of precipitation as random effects.

We assessed the effect of microhabitat including shrub characteristics, grass height, herbaceous canopy cover, and ground cover variables on nest success (Table 2). In addition to the microhabitat variables, we compared nest success within and outside of Core Areas with a categorical variable (Cor_NonCore). All possible combinations of informative variables were included as additive models and compared with AIC_*c*_ and *w*_*i*_. Nest success estimates were calculated with the Kaplan-Meier product-limit estimator [47].

## Results

We obtained microhabitat samples at 928 nests and 819 random locations across study areas from 2008 to 2014 (Table 1). All nests and random locations (*n* = 1,747) were used in the nest-random analysis, 819 random locations were used in the random-random analysis, and 928 nests were used in the nest-nest analysis. We recorded information on nest success at 924 nests, which were used in nest success analysis. Microhabitat sampling locations were predominantly in Core Areas where 82% of nests and 73% of random locations occurred.

### Nest-Random

Competitive models that best explained sage-grouse microhabitat nest selection included 12 predictor variables that described shrub characteristics, grass height, herbaceous canopy cover, and ground cover microhabitat characteristics (Table 3). In the nest-random modeling set, 73 models were competitive with the top model (ΔAIC_*c*_ = 0.25–3.96). The global model (K = 15, ΔAIC_*c*_ = 10.25) was not in the competitive model set. Model averaging indicated that the 95% confidence interval for the odds ratio estimate of Gap_3m_, PerGrass_H, Grass_5m_, Forb_1m_, BioCrust_5m_, and Litter_5m_ overlapped 1 (Table 4); therefore, we considered those to be marginal predictor variables and limited our interpretations to primarily focus on the Shrub_1m_, Artr_2.5m_, Artr_H, Gap_0.5m_, V0, and BareRock_5m_ variables. Supported microhabitat variables encompassed all three microhabitat scales (1-m, 2.5-m, and 5-m). For every 10% increase in shrub cover at the 1-m scale (Shrub_1m_), the relative probability of nest selection increased by approximately 48%. A 10% increase in big sagebrush cover within 2.5 m (Artr_2.5m_) resulted in an increase in relative probability of nest selection by approximately 23%. For every 1 unit increase in the number of spaces between shrubs at the plot scale (≥0.5 m and <1.0 m; Gap_0.5m_) relative probability of nest selection increased by approximately 8%. Increased visual obstruction (VO) by 10 cm increased the relatively probability of nest selection by 28%.

**Table 3:** Model comparisons of binomial generalized linear mixed models evaluating nest-site selection of sage-grouse (nest-random), availability of microhabitat within and outside of Core Areas (random-random), and sage-grouse use of microhabitat within and outside of Core Areas (nest-nest). Cox proportional hazard models were used to evaluate nest success of sage-grouse within and outside of Core Areas. Top five models for each analysis type were compared with Akaike’s information criterion (adjusted for small sample sizes; AIC_*c*_) and Akaike weights (*w*_*i*_). Nests and random locations were located in five distinct study areas (*n* = 928 nests, *n* = 819 random locations, and *n* = 924 nests with survival data) throughout central and southwestern Wyoming, USA, 2008–2014.

	Model fit statistics			
Model^1^	K	ΔAIC	*w*_*i*_	Deviance
**Nest-random**				
Shrub_1m_ + Artr_2.5m_ + Artr_H + Gap_0.5m_ + VO + BareRock_5m_	9	0.00	0.03	1965.12
Shrub_1m_ + Artr_2.5m_ + Artr_H + Gap_0.5m_ + VO	8	0.25	0.03	1967.39
Shrub_1m_ + Artr_2.5m_ + Gap_0.5m_ + VO + BareRock_5m_	8	0.56	0.03	1967.70
Shrub_1m_ + Artr_2.5m_ + Gap_0.5m_ + VO	7	1.07	0.02	1970.23
Shrub_1m_ + Artr_2.5m_ + Artr_H + Gap_0.5m_ + Gap_3m_ + VO + BareRock_5m_	10	1.25	0.02	1964.34
Null AIC_*c*_ = 400.13				
**Random-random**				
Gap_0.5m_ + ResGrass_1m_ + Cactus_1m_ + BioCrust_2.5–3.5m_ + Litter_5m_	8	0.00	0.22	428.30
Gap_0.5m_ + ResGrass_1m_ + Cactus_1m_	6	0.35	0.19	432.72
Gap_0.5m_ + ResGrass_1m_ + Cactus_1m_ + BioCrust_2.5–3.5m_	7	0.72	0.15	431.06
Gap_0.5m_ + ResGrass_1m_ + Cactus_1m_ + Litter_5m_	7	1.28	0.12	431.62
Gap_0.5m_ + ResGrass_1m_ + BioCrust_2.5–3.5m_ + Litter_5m_	7	1.82	0.09	432.16
Null AIC_*c*_ = 14.12				
**Nest-nest**				
Artr_H + Gap_0.5m_ + Bare_5m_	6	0.00	0.06	373.98
Artr_H + Gap_0.5m_	5	0.01	0.06	376.02
Artr_H + Gap_0.5m_ + Bare_5m_ + Rock_5m_	7	0.07	0.06	372.02
Artr_H + Bare_5m_ + Rock_5m_	6	0.57	0.05	374.54
Artr_H + Gap_0.5m_ + Rock_5m_	6	0.62	0.05	374.60
Null AIC_*c*_ = 3.30				
**Nest success**				
ARTRpShr_2.5m_ + Gap_2m_ + Rock_2.5–3.5m_	5	0.00	0.54	6056.86
ARTRpShr_2.5m_ + Gap_2m_	4	2.40	0.16	6063.43
Gap_2m_ + Rock_2.5–3.5m_	4	3.11	0.11	6057.00
ARTRpShr_2.5m_ + Gap_2m_	4	3.55	0.09	6054.18
Gap_2m_	3	4.60	0.05	6054.12
Null AIC_*c*_ = 9.74				

^1^Only the top five models and the null model are reported for each analysis type.

**Table 4:** Model-averaged parameter estimates and odds ratios with 95% confidence intervals (CI). Data were collected from five distinct study areas (*n* = 928 nests and *n* = 819 random locations) in central and southwestern Wyoming, USA, 2008–2014.

			95% CI			95% CI	
Parameter	Estimate	SE	Lower	Upper	Odds ratio	Lower	Upper
**Nest-random**							
Intercept	3.92						
Shrub_1m_	0.04	0.01	0.03	0.05*	1.04	1.03	1.05*
Artr_2.5m_	0.02	0.01	0.01	0.03*	1.02	1.01	1.03*
Artr_H	0.01	0.01	0.00	0.02*	1.01	1.00	1.02*
Gap_0.5m_	0.08	0.01	0.05	0.11*	1.08	1.05	1.12*
Gap_3m_	-0.08	0.08	-0.26	0.10	0.92	0.77	1.10
VO	0.02	0.01	0.01	0.03*	1.02	1.01	1.03*
PerGrass_H	0.01	0.01	-0.01	0.02	1.01	0.99	1.02
Grass_5m_	-0.00	0.01	-0.02	0.02	1.00	0.98	1.02
Forb_1m_	-0.00	0.01	-0.02	0.02	1.00	0.98	1.02
BareRock_5m_	-0.01	0.01	-0.02	0.00*	0.99	0.98	1.00*
BioCrust_5m_	0.01	0.02	-0.03	0.05	1.01	0.98	1.05
Litter_5m_	0.00	0.01	-0.01	0.01	1.00	0.99	1.01
**Random-random**							
Intercept	10.57						
Gap_0.5m_	0.38	0.10	0.19	0.56*	1.46	1.22	1.76*
ResGrass_1m_	-0.16	0.04	-0.24	-0.07*	0.86	0.79	0.93*
Cactus_1m_	-0.69	0.31	-1.30	-0.08*	0.50	0.27	0.92*
BioCrust_2.5–3.5m_	0.17	0.08	0.01	0.34*	1.19	1.01	1.40*
Litter_5m_	-0.03	0.02	-0.07	0.00*	0.97	0.94	1.00*
**Nest-nest**							
Intercept	10.29						
Artr_H	-0.03	0.02	-0.06	0.00*	0.97	0.94	1.00*
Gap_0.5m_	0.16	0.10	-0.03	0.35	1.17	0.97	1.42
AnGrass	-0.08	0.06	-0.19	0.04	0.93	0.83	1.04
Bare_5m_	0.06	0.04	-0.02	0.13	1.06	0.98	1.14
Rock_5m_	-0.04	0.03	-0.10	0.02	0.96	0.90	1.02
**Nest Success**							
Gap_2m_	-0.06	0.04	-013	0.01	0.95	0.87	1.01
ARTRpShr_2.5m_	0.30	0.19	-0.07	0.67	1.35	0.93	1.96
Rock_2.5–3.5m_	-0.01	0.01	-0.02	0.00*	0.99	0.98	1.00*

* Indicates 95% CI not overlapping 0 for beta estimates or 1 for odds ratios

### Random-Random

Models that best explained microhabitat availability between Core and Non-Core Areas included 5 predictor variables (Table 3). The candidate model set consisted of 7 models (ΔAIC_*c*_ = 0.35–3.48). Model averaging indicated that the 95% confidence intervals for odds ratio estimates of the 5 predictor variables did not overlap 1 (Table 4). Therefore, our top model explaining microhabitat availability included the variables Gap_0.5m_, ResGrass_1m_, Cactus_1m_, BioCrust_2.5–3.5m_, and Litter_5m_ at all 3 microhabitat scales. The number of shrub gap spaces (≥0.5 m and <1.0 m; Gap_0.5m_) and biological soil crust between 2.5 and 3.5 m away from the center of the transect (BioCrust_2.5–3.5m_) were strong positive predictors of microhabitat availability in Core Areas. Residual grass cover within 1 m (ResGrass_1m_) of center of random locations, cactus cover between 2.5 and 3.5 m away from the center of the transect, and litter within 5 m away from the transect center (Litter_5m_) were all negative predictors of microhabitat availability in Core Areas. We report means and SE for microhabitat data at random locations within and outside of Core Areas nest-nest in S2 Table.

### Nest-Nest

The models that explained nest site selection between Core and Non-Core Areas included 5 predictor variables across 31 models, including the null model (ΔAIC_*c*_ = 0.01–3.30). The null model was competitive with our top model—within 4 ΔAIC_*c*_, which indicated that the top model was not much better at explaining differences between Core and Non-Core Area nests than the null model. The global model (K = 8, ΔAIC_*c*_ = 16.00) was not in the competitive model set. Our top model comparing microhabitat differences between Core and Non-Core Areas included the variables Artr_H, Gap_0.5m_, and Bare_5m_. Model averaging indicated that the 95% confidence intervals for the odds ratio estimate of Artr_H did not overlap 1 (Table 4). Nests in Core Areas tended to have lower mean sagebrush height within 5 m of those nests compared to nests in Non-Core Areas. Model averaging indicated that the 95% confidence interval for the odds ratio estimates of Gap_0.5m_, AnGrass, Bare_5m_, and Rock_5m_ overlapped 1 (Table 4); therefore, we considered those to be marginal predictor variables. We report means and SE for microhabitat data at nests within and outside of Core Areas nest-nest in S3 Table.

### Nest Success

Average annual apparent nest success for all nests was 48.1% (444 hatched nests of 924), and the Kaplan-Meier nest success estimate for a 27-day incubation period was 42.0% (95% CI: 38.4–45.9%). No microhabitat variables in our final models violated the proportional hazards assumption of the Cox proportional hazards model. We did not find a difference in nest success between nests within and outside of Core Areas.

The top model describing nest success of sage-grouse included ARTRpShr_2.5m_, Gap_2m_, and Rock_2.5–3.5m_. There were three other competitive models within ΔAIC_*c*_ = 2.40–3.55 that included these variables (Table 3); thus, we model-averaged coefficient estimates. Model averaging indicated that Rock_2.5–3.5m_ was the only variable to have model-averaged 95% CI of odds ratios that did not overlap 1 (Table 4). Although Gap_2m_ and ARTRpShr_2.5m_ were considered in additive modeling, those variables were marginal predictors with model-averaged 95% CI of odds ratios overlapping 1 (Table 4). Therefore, we limited our interpretations to primarily focus on the Rock_2.5–3.5m_ variable (Table 4). The top model included all variables considered in additive modeling, but was not the global model (i.e., model including all hypothesized variables). The null model was not competitive (ΔAIC_*c*_ = 9.74) with models used in model averaging (Table 3). Rock cover between 2.5 and 3.5 m away from a nest (Rock_2.5–3.5m_) was negatively associated with failure; thus, greater rock ground cover was associated with higher nest success (Table 4). We report means and SE for microhabitat data at successful and unsuccessful nests in S4 Table.

## Discussion

Similar to other studies, we found that sage-grouse selected nesting habitat with greater concealment cover including sagebrush cover and height, and visual obstruction [7,9,14,15]. We found a positive association between nest selection and perennial grass height; however, this relationship had marginal support. Microhabitat characteristics associated with less bare ground and rock cover were not selected for compared to random locations. Females selected for more small gaps between shrubs (gap sizes ≥0.5 m and <1.0 m; Gap_0.5m_). Locations with more small gaps but few large gaps may indicate 1) areas with greater homogeneity in shrub cover with more sagebrush distributed across the plot (i.e., no large holes in cover) or 2) provide adequate concealment while simultaneously allowing the female to detect and escape from predators.

From our random-random analyses, the availability of small gaps between shrubs was the only shrub variable that differed within and outside of Core Areas, with Core Areas having greater availability of small gaps. Core Areas had lower availability of residual grass and litter cover. This may be related to greater livestock grazing in these areas [e.g., 48], but we do not have data to support this and Wyoming’s Core Area Policy does not include any measures that alter livestock grazing in Core Areas. Randomly available locations in Core Areas had more biological soil crust indicating areas with better soil stability. However, biological soil crusts can be compromised by excessive livestock trampling, especially during the growing season when crusts are not frozen [49], suggesting that grazing may not be reducing residual grass and litter in Core Areas compared to nearby non-Core Areas.

Sage-grouse use of microhabitat within and outside of Core Areas (nest-nest analysis) indicated that nests within Core Areas had lower big sagebrush height. Sage-grouse nests within Core Areas had shorter sagebrush heights, which may be associated with differing composition of sagebrush species. For instance, sage-grouse nests in the Jeffrey City study area primarily occurred in Core Area in Wyoming big sagebrush communities, whereas nests in the Bighorn Basin study area often occurred outside of Core Areas in taller mountain big sagebrush communities. We found little support to suggest that annual grass, bare ground, and rock cover were predictive of nests within and outside Core Areas. Albeit, annual grasses are an important indicator of the resistance and resilience of sage-grouse habitats to respond to disturbance [50]. In addition, nest sites selected by sage-grouse in the Atlantic Rim and Stewart Creek of south-central Wyoming were negatively correlated with the presence of cheatgrass (*Bromus tectorum*), but positively correlated with greater perennial grass cover, litter, sagebrush cover, and visual obstruction [7].

Most of our microhabitat sampling locations were in Core Areas (82% of nests and 73% of random locations); however, we found little supporting evidence that Wyoming’s Core Area Policy increased the availability of quality microhabitat for sage-grouse between 2008 and 2014. Because the Core Area Policy is relatively new and the primary focus of this policy is on landscape scale conservation—such as limits on oil and gas development—and not on anthropogenic activities more likely to affect microhabitat conditions such as grazing, it is not surprising that we only detected a slight difference. We found some differences in microhabitat characteristics within and outside Core Areas, but these differences were relatively minor relative to a landscape scale conservations policy. Also, our microhabitat sampling locations outside of Core Areas were adjacent to Core Areas (mean distance from Core Areas = 3.1 km [SD = 2.3] km with range of 0.04–12.0 km). Many of our locations outside of Core Areas had not been subject to greater surface disturbance than locations within Core Areas, and we did not continually monitor in landscapes being actively developed for more than 4 years. Thus, our sampling locations (nest and random locations) within and outside of Core Areas were generally in similar habitats.

The connection between specific microhabitat characteristics and nest success has not been consistently documented. Our nest success results need to be interpreted as effects of microhabitat characteristics within the range of what sage-grouse selected compared to what was available to them. Rock cover in our study areas was composed mainly of small diameter gravel <3 cm. It is possible nest placement within areas of greater rock ground cover may be a conferred adaptive advantage related to female concealment during incubation. Female sage-grouse have cryptic grayish-brown plumage [51] that may conform to nesting areas with a high percent of rock ground cover, which could lead to a lower probability of being discovered by visual predators. Nests with greater rock ground cover could also be correlated with areas closer to ridgelines that tend to be less traveled by olfactory predators, which tend to utilize drainage bottoms [52].

Although a marginal finding, we found that nests with greater heterogeneity in shrub species within 2.5 m of a nest were more successful, which could be related to areas with greater vegetative productivity. While most sage-grouse nests are found under sagebrush species [17], Musil [53] found that sage-grouse used shrub species other than big sagebrush more than expected in Idaho. Contrary to our expectations, sage-grouse had higher nest success at nests with more ≥2 m and <3.0 m (Gap_2m_) gaps between shrubs within 5 m of the nest and more rock cover between 2.5 and 3.5 m away from a nest; however, this variable was a marginal finding with 95% CI of odds ratios overlapping 1. This finding is bounded by our results illustrating that sage-grouse selected areas with fewer large gaps. The olfactory ability of mammals is impeded by more updrafts and greater wind turbulence at the microhabitat-scale, and both of these climatic conditions are more likely to occur in areas with local heterogeneity in vegetation height [52]. Conover et al. [54] did not find any differences in updrafts or wind turbulence between nest and random or successful and unsuccessful sage-grouse nests. However, they only tested wind conditions directly above the nest or random location. There may be a nest success trade-off between areas with greater visual obstruction (where sage-grouse typically choose to place their nests) and areas with better olfactory obstruction near the nest (nests with more medium sized gaps within 5 m of the nest that create local updrafts and allow for higher wind velocities near the nest); however, this is an untested hypothesis. Alternatively, there may be a potential correlation of this size gap with a microhabitat characteristic that was not measured (e.g., micro-scale topography, etc.).

Although general trends did emerge, we found few differences between microhabitat selection by sage-grouse within and outside of Core Areas, and no difference in nest success. Sage-grouse selected nests with greater concealment cover, and we found little evidence to suggest that there were differences in concealment cover at available habitats or nest sites in and outside of the Core Areas that were assessed. In addition, we found no evidence that concealment cover was important for nest success in our study areas. The Core Area Policy was designed to reduce negative impacts to quality sage-grouse breeding habitat [21], but our results suggest that the policy may primarily operate at larger spatial scales. As resource selection is a function of available habitats [55], changes in habitat quality may be manifested through management practices resulting in vegetative changes through time. Sage-grouse are a landscape species, with studies demonstrating that both nest selection and success are influenced by habitat characteristics at multiple spatial scales [6,16,56,57]. It is possible that changes in habitat quality at larger spatial scales would eventually have cascading effects on microhabitat quality. Continued assessment of differences in available habitat and nest success will be important steps to assess the viability of Wyoming’s Core Area Policy. We found strong evidence that conservation of sage-grouse needs to maintain the availability of concealment cover at the microhabitat spatial scale for nesting. The inclusion of microhabitat information in sage-grouse conservation strategies will help management agencies better understand the relationships between fine scale microhabitat attributes and how those attributes influence sage-grouse nest selection, nest success, and ultimately habitat quality.

## Supporting Information

S1 Table Nest-random microhabitat data. Mean habitat characteristics (± SE) sampled within 5 m of nest and random locations for nest-random comparison in 5 study areas in central and southwestern Wyoming, USA, 2008–2014.
(DOCX)

S2 Table Nest-random microhabitat data. Mean habitat characteristics (± SE) sampled within 5 m of nest and random locations for nest-random comparisons in 5 study areas in central and southwestern Wyoming, USA, 2008–2014.
(DOCX)

S3 Table Nest-random microhabitat data. Mean habitat characteristics (± SE) sampled within 5 m of nest locations within and outside of Core Areas for nest-nest comparisons in 5 study areas in central and southwestern Wyoming, USA, 2008–2014.
(DOCX)

S4 Table Nest-random microhabitat data. Mean habitat characteristics (± SE) sampled within 5 m of successful and unsuccessful nest locations in 5 study areas in central and southwestern Wyoming, USA, 2008–2014.
(DOCX)

S5 Table Nest and random location microhabitat data. Microhabitat data for random and nest locations. Nest data includes data on nest survival.
(CSV)
