# Supplementary material for: Microhabitat Conditions in Wyoming’s Sage-Grouse Core Areas: Effects on Nest Site Selection and Success
Source: PLoS One. 2016 Mar 22;11(3):e0150798. doi: 10.1371/journal.pone.0150798 (PMC4803343; doi:10.1371/journal.pone.0150798)
Supplement: S1 Table — Mean habitat characteristics (± SE) sampled within 5 m of nest and random locations for nest-random comparison in 5 study areas in central and southwestern Wyoming, USA, 2008–2014. (DOCX) [file pone.0150798.s001.docx]

**S1 Table.** Mean habitat characteristics (± SE) sampled within 5 m of nest and random locations for nest-random comparisons in 5 study areas in central and southwestern Wyoming, USA, 2008–2014.

|  | Nests | | Random | |
| --- | --- | --- | --- | --- |
| Habitat characteristic | Mean | SE | Mean | SE SE |
| **Shrub characteristics** |  |  |  |  |
| Shrub | 36.69 | 0.51 | 26.13 | 0.48 |
| Artr | 29.25 | 0.50 | 19.06 | 0.45 |
| Shrub_H | 39.85 | 0.50 | 34.18 | 0.56 |
| Artr_H | 40.83 | 0.56 | 32.81 | 0.63 |
| VO | 35.84 | 0.51 | 25.95 | 0.45 |
| **Grass Height** |  |  |  |  |
| PerGrass_H | 29.88 | 0.37 | 25.58 | 0.38 |
| ResGrass_H | 17.73 | 0.21 | 16.29 | 0.23 |
| **Herbaceous Canopy Cover (%)** |  |  |  |  |
| AnGrass | 2.14 | 0.11 | 1.75 | 0.10 |
| PerGrass | 14.2 | 0.19 | 13.51 | 0.19 |
| ResGrass | 7.52 | 0.13 | 7.17 | 0.14 |
| FoodF | 5.19 | 0.12 | 5.01 | 0.13 |
| NFoodF | 2.38 | 0.08 | 2.24 | 0.08 |
| **Ground Cover (%)** |  |  |  |  |
| BGround | 25.82 | 0.31 | 27.03 | 0.36 |
| Cactus | 0.37 | 0.04 | 0.30 | 0.04 |
| BioCrust | 3.31 | 0.12 | 2.80 | 0.11 |
| Rock | 8.44 | 0.20 | 8.68 | 0.20 |
| Litter | 40.31 | 0.38 | 38.4 | 0.43 |
|  |  |  |  |  |
