# Supplementary material for: Microhabitat Conditions in Wyoming’s Sage-Grouse Core Areas: Effects on Nest Site Selection and Success
Source: PLoS One. 2016 Mar 22;11(3):e0150798. doi: 10.1371/journal.pone.0150798 (PMC4803343; doi:10.1371/journal.pone.0150798)
Supplement: S2 Table — Mean habitat characteristics (± SE) sampled within 5 m of nest and random locations for nest-random comparisons in 5 study areas in central and southwestern Wyoming, USA, 2008–2014. (DOCX) [file pone.0150798.s002.docx]

**S2 Table.** Mean habitat characteristics (± SE) sampled within 5 m of random locations within and outside of Core Areas for random-random comparisons in 5 study areas in central and southwestern Wyoming, USA, 2008–2014.

|  | Core | | Non-Core | |
| --- | --- | --- | --- | --- |
| Habitat characteristic | Mean | SE | Mean | SE SE |
| **Shrub characteristics** |  |  |  |  |
| Shrub | 26.35 | 0.56 | 25.56 | 0.96 |
| Artr | 19.37 | 0.52 | 18.20 | 0.90 |
| Shrub_H | 32.52 | 0.59 | 38.64 | 1.27 |
| Artr_H | 31.54 | 0.72 | 36.23 | 1.29 |
| VO | 25.37 | 0.51 | 27.49 | 0.96 |
| **Grass Height** |  |  |  |  |
| PerGrass_H | 25.67 | 0.42 | 25.35 | 0.81 |
| ResGrass_H | 16.46 | 0.26 | 15.82 | 0.45 |
| **Herbaceous Canopy Cover (%)** |  |  |  |  |
| AnGrass | 1.75 | 0.12 | 1.76 | 0.20 |
| PerGrass | 13.59 | 0.22 | 13.28 | 0.40 |
| ResGrass | 7.23 | 0.17 | 7.01 | 0.27 |
| FoodF | 5.06 | 0.16 | 4.86 | 0.24 |
| NFoodF | 2.27 | 0.09 | 2.14 | 0.15 |
| **Ground Cover (%)** |  |  |  |  |
| BGround | 26.74 | 0.43 | 27.83 | 0.67 |
| Cactus | 0.27 | 0.04 | 0.37 | 0.09 |
| BioCrust | 2.77 | 0.13 | 2.90 | 0.23 |
| Rock | 8.81 | 0.24 | 8.32 | 0.39 |
| Litter | 38.31 | 0.50 | 38.64 | 0.86 |
|  |  |  |  |  |
