# Supplementary material for: Microhabitat Conditions in Wyoming’s Sage-Grouse Core Areas: Effects on Nest Site Selection and Success
Source: PLoS One. 2016 Mar 22;11(3):e0150798. doi: 10.1371/journal.pone.0150798 (PMC4803343; doi:10.1371/journal.pone.0150798)
Supplement: S3 Table — Mean habitat characteristics (± SE) sampled within 5 m of nest locations within and outside of Core Areas for nest-nest comparisons in 5 study areas in central and southwestern Wyoming, USA, 2008–2014. (DOCX) [file pone.0150798.s003.docx]

**S3 Table.** Mean habitat characteristics (± SE) sampled within 5 m of nest locations within and outside of Core Areas for nest-nest comparisons in 5 study areas in central and southwestern Wyoming, USA, 2008–2014.

|  | Core | | Non-Core | |
| --- | --- | --- | --- | --- |
| Habitat characteristic | Mean | SE | Mean | SE SE |
| **Shrub characteristics** |  |  |  |  |
| Shrub | 36.64 | 0.56 | 36.92 | 1.22 |
| Artr | 29.05 | 0.54 | 30.19 | 1.24 |
| Shrub_H | 38.34 | 0.52 | 46.77 | 1.30 |
| Artr_H | 39.64 | 0.60 | 46.30 | 1.32 |
| VO | 35.34 | 0.55 | 38.16 | 1.33 |
| **Grass Height** |  |  |  |  |
| PerGrass_H | 29.19 | 0.39 | 33.08 | 1.00 |
| ResGrass_H | 17.51 | 0.23 | 18.76 | 0.50 |
| **Herbaceous Canopy Cover (%)** |  |  |  |  |
| AnGrass | 2.17 | 0.12 | 2.02 | 0.23 |
| PerGrass | 13.99 | 0.20 | 15.19 | 0.47 |
| ResGrass | 7.44 | 0.14 | 7.89 | 0.33 |
| FoodF | 5.15 | 0.14 | 5.40 | 0.31 |
| NFoodF | 2.36 | 0.09 | 2.49 | 0.20 |
| **Ground Cover (%)** |  |  |  |  |
| BGround | 25.84 | 0.34 | 25.75 | 0.76 |
| Cactus | 0.35 | 0.04 | 0.43 | 0.09 |
| BioCrust | 3.17 | 0.12 | 3.93 | 0.32 |
| Rock | 8.62 | 0.22 | 7.62 | 0.43 |
| Litter | 40.22 | 0.42 | 40.69 | 0.90 |
|  |  |  |  |  |
