# Supplementary material for: Microhabitat Conditions in Wyoming’s Sage-Grouse Core Areas: Effects on Nest Site Selection and Success
Source: PLoS One. 2016 Mar 22;11(3):e0150798. doi: 10.1371/journal.pone.0150798 (PMC4803343; doi:10.1371/journal.pone.0150798)
Supplement: S4 Table — Mean habitat characteristics (± SE) sampled within 5 m of successful and unsuccessful nest locations in 5 study areas in central and southwestern Wyoming, USA, 2008–2014. (DOCX) [file pone.0150798.s004.docx]

**S4 Table.** Mean habitat characteristics (± SE) sampled within 5 m of successful and unsuccessful nest locations in 5 study areas in central and southwestern Wyoming, USA, 2008–2014.

|  | Successful | | Unsuccessful | |
| --- | --- | --- | --- | --- |
| Habitat characteristic | Mean | SE | Mean | SE SE |
| **Shrub characteristics** |  |  |  |  |
| Shrub | 36.95 | 0.74 | 36.52 | 0.71 |
| Artr | 28.73 | 0.72 | 29.74 | 0.69 |
| Shrub_H | 39.15 | 0.70 | 40.51 | 0.71 |
| Artr_H | 39.94 | 0.81 | 41.66 | 0.77 |
| VO | 35.94 | 0.77 | 35.85 | 0.68 |
| **Grass Height** |  |  |  |  |
| PerGrass_H | 29.85 | 0.55 | 29.89 | 0.49 |
| ResGrass_H | 17.81 | 0.30 | 17.64 | 0.29 |
| **Herbaceous Canopy Cover (%)** |  |  |  |  |
| AnGrass | 2.23 | 0.16 | 2.06 | 0.14 |
| PerGrass | 13.98 | 0.27 | 14.39 | 0.26 |
| ResGrass | 7.46 | 0.19 | 7.56 | 0.18 |
| FoodF | 5.03 | 0.18 | 5.33 | 0.17 |
| NFoodF | 2.46 | 0.12 | 2.32 | 0.11 |
| **Ground Cover (%)** |  |  |  |  |
| BGround | 25.80 | 0.45 | 25.91 | 0.42 |
| Cactus | 0.38 | 0.06 | 0.35 | 0.05 |
| BioCrust | 3.30 | 0.17 | 3.31 | 0.16 |
| Rock | 8.66 | 0.29 | 8.28 | 0.27 |
| Litter | 40.30 | 0.53 | 40.23 | 0.54 |
|  |  |  |  |  |
